# Effectiveness of Brief Behavioral Intervention for Insomnia on Anxiety, Depression, Quality of Life, and Sleep: A Randomized Pilot Trial

**DOI:** 10.7759/cureus.111676

**Published:** 2026-06-28

**Authors:** Horacio Balam Alvarez Garcia, Ulises Jimenez Correa, Alejandro Jiménez Genchi

**Affiliations:** 1 Master's and Doctoral Program in Medical, Dental, and Health Sciences, Faculty of Medicine, Universidad Nacional Autónoma de México, Mexico City, MEX; 2 Sleep Disorders Clinic/Research Division, Faculty of Medicine, Universidad Nacional Autónoma de México, Mexico City, MEX; 3 Sleep Clinic, Instituto Nacional de Psiquiatría Ramón de la Fuente Muñiz, Mexico City, MEX

**Keywords:** covid-19, insomnia, mental health, quality of life, telemedicine

## Abstract

Introduction

Sleep disorders and psychiatric diseases are public health problems that have increased due to the COVID-19 lockdown. In Mexico, telemedicine was a frequently used treatment modality under the circumstances of the lockdown. The present study aimed to test the effectiveness of brief behavioral intervention for improving symptoms of insomnia, anxiety, and depression and increasing quality of life in patients who survived the COVID-19 infection.

Method

A randomized, parallel-group equivalence clinical trial was conducted without a passive control group. Pre-treatment, post-treatment, and three-month follow-up measurements were taken. The intervention consisted of four weekly sessions lasting 50 minutes each. Five questionnaires were applied: Insomnia Severity Index (ISI), Pittsburgh Sleep Quality Index (PSQI), General Anxiety Disorder 7 (GAD-7), Patient Health Questionnaire 9 (PHQ-9), SF-36 Health Questionnaire (SF36), and a sleep diary.

Results

In both therapy modalities, significant improvement was identified (p < 0.05) in ISI (14.57 vs 5.57), ICSP (12.43 vs 4.86), GAD-7 (13.00 vs 5.29), PHQ-9 (15.86 vs 5.00), and SF-36 (30.00 vs 77.14) scores, which coincided with sleep diary measurements.

Conclusion

Both intervention modalities were effective, indicating that teleconsultation is a viable treatment option for insomnia symptoms and mental health comorbidities.

## Introduction

During the COVID-19 pandemic, telemedicine became the most widely used treatment modality due to the multiple benefits involved, especially in terms of reducing the risk of contagion during the lockdown [1]. In addition, it has been observed that online therapy is effective for some mental disorders beyond the context of the pandemic [2]. In sleep medicine, telemedicine has shown effectiveness for the management of disorders such as insomnia and obstructive sleep apnea, both in the general population and in COVID-19 survivors [3-5].

Telemedicine applied to sleep disorders has focused on cognitive behavioral therapy for insomnia (CBT-I) and therapeutic adherence to the continuous positive airway pressure (CPAP) device [6,7]. CBT-I is a structured treatment of four to eight sessions in which behavioral techniques (sleep restriction, stimulus control therapy, progressive muscle relaxation), cognitive techniques (cognitive restructuring, paradoxical intention, attentional control), and educational techniques (sleep hygiene) are applied with the aim of controlling the triggers and maintenance of insomnia [8]. This treatment has been shown to be effective in various teleconsultation modalities (live video, remote patient monitoring, or mobile applications) and has therefore been proposed as the first-choice treatment for insomnia [9-12].

In developing countries, research related to tele-health effectiveness has begun to gain importance due to the advantages that this modality presents in public health services, such as the reduction in patient commuting and providing health services to remote communities [13], as well as a decrease in costs. In the case of Mexico, it has been observed that teleconsultation has been effective for addressing psychiatric symptoms such as anxiety and depression [14]. However, no randomized clinical trials (RCTs) of any kind (pilot, equivalence, superiority, or non-inferiority) have been carried out to evaluate the effectiveness of teleconsultation for patients with insomnia and psychiatric symptomatology such as anxiety, depression, or post-traumatic stress disorder [15], representing a knowledge gap [16].

A high prevalence and persistence of insomnia symptoms have been reported in individuals who survived SARS-CoV-2 infection. In a study by Kyzar et al., the prevalence was estimated at 50% that decreased slightly to 42% at a second evaluation, with more severe symptoms of anxiety and depression strongly associated with higher scores on the Insomnia Severity Index (ISI) [17]. In this context, it is necessary to address insomnia symptoms in patients who have recovered from SARS-CoV-2, many of whom were exposed to significant stress or trauma during the illness, to prevent the potential progression of symptoms to more severe conditions such as post-traumatic stress disorder (PTSD) [18].

The aim of this study was, therefore, to test the effectiveness of the Brief Behavioral Intervention for Insomnia (BBII) administered in traditional face-to-face form or by tele-consultation, on symptoms of insomnia, anxiety, depression, and quality of life of patients who survived COVID-19 in Mexico City. The working hypothesis was that BBII would be effective in reducing symptoms of insomnia, anxiety, and depression, as well as improving quality of life in both administration modalities. This was based on trends in the literature indicating that teleconsultation modalities have had good results in multiple clinical, equivalence, and non-inferiority trials.

## Materials and methods

This was a randomized, parallel-group equivalence clinical trial without a passive control group. Pre-treatment, post-treatment, and three-month follow-up data were collected. The study was approved by the Ethics Committee of Hospital General de Mexico "Dr. Eduardo Liceaga" (approval number: DI/23/UME/03/9) and registered at clinicaltrials.gov (NCT05951803) [19].

Study population

Eligibility Criteria

Participants were recruited from the Hospital General de Mexico "Dr. Eduardo Liceaga" in Mexico City, Mexico, primarily from areas with a high incidence of patients presenting with insomnia, depression, or anxiety. The sample consisted mainly of family members of hospitalized patients who visited the clinic for insomnia symptoms. During the initial interview, the severity of the symptoms was assessed, and if necessary, referrals were made to a highly specialized service (such as psychiatry) within the same hospital.

The inclusion criteria were: (i) Medical history of COVID-19 diagnosed by polymerase chain reaction (PCR) or antigen test, (ii) Mild, moderate, symptoms of insomnia, anxiety, or depression (but without a psychiatric diagnosis of anxiety or depression), (iii) Age between 18 and 40 years, and (iv) Access to a computer, tablet, or smartphone with internet service.

The exclusion criteria included: (i) Psychological treatment for insomnia, anxiety, or depression during the study, (ii) Cardiorespiratory or neurological sequelae due to COVID-19 that would prevent the participant from taking the BBII test, (iii) Symptoms of another sleep disorder, such as obstructive sleep apnea, restless legs syndrome, or shift work sleep disorder, and (iv) Use of addictive substances (such as tobacco, cannabis, or cocaine). 

Finally, the withdrawal criteria used were the following: (i) Desire to discontinue BBII therapy, (ii) Missed two consecutive treatment sessions, and (iii) Started pharmacological treatment for anxiety, depression, or insomnia.

Sample Size

The originally estimated sample size was 34 participants; however, only 17 met all inclusion criteria and provided informed consent. The analysis was thus performed using the available sample, following the recommendation of the supervising research team. Given the pilot nature of this study, formal justification of the sample size based on statistical power was not the primary criterion; rather, the sample size was justified for feasibility reasons, as pilot trials are designed to assess the feasibility of recruitment procedures, protocol adherence, and to generate preliminary effect size estimates that can inform the design of future, fully powered equivalence trials.

Interventions

BBII was implemented in two different modalities: (i) teleconsultation (BBII-TC), and (ii) traditional in-person sessions (BBII-FF). Participants in the BBII-TC group received four treatment sessions in which they were instructed on the techniques described above. Each session lasted 50 minutes and was scheduled weekly via the institutional platform Zoom (Zoom Communications, Inc., San Jose, California, United States) to ensure session privacy. On the other hand, participants in the BBII-FF received four face-to-face treatment sessions. In these sessions, they were instructed on the techniques described above. Each session lasted 50 minutes. In both groups, the weekly sessions were conducted individually. 

**Figure 1:**
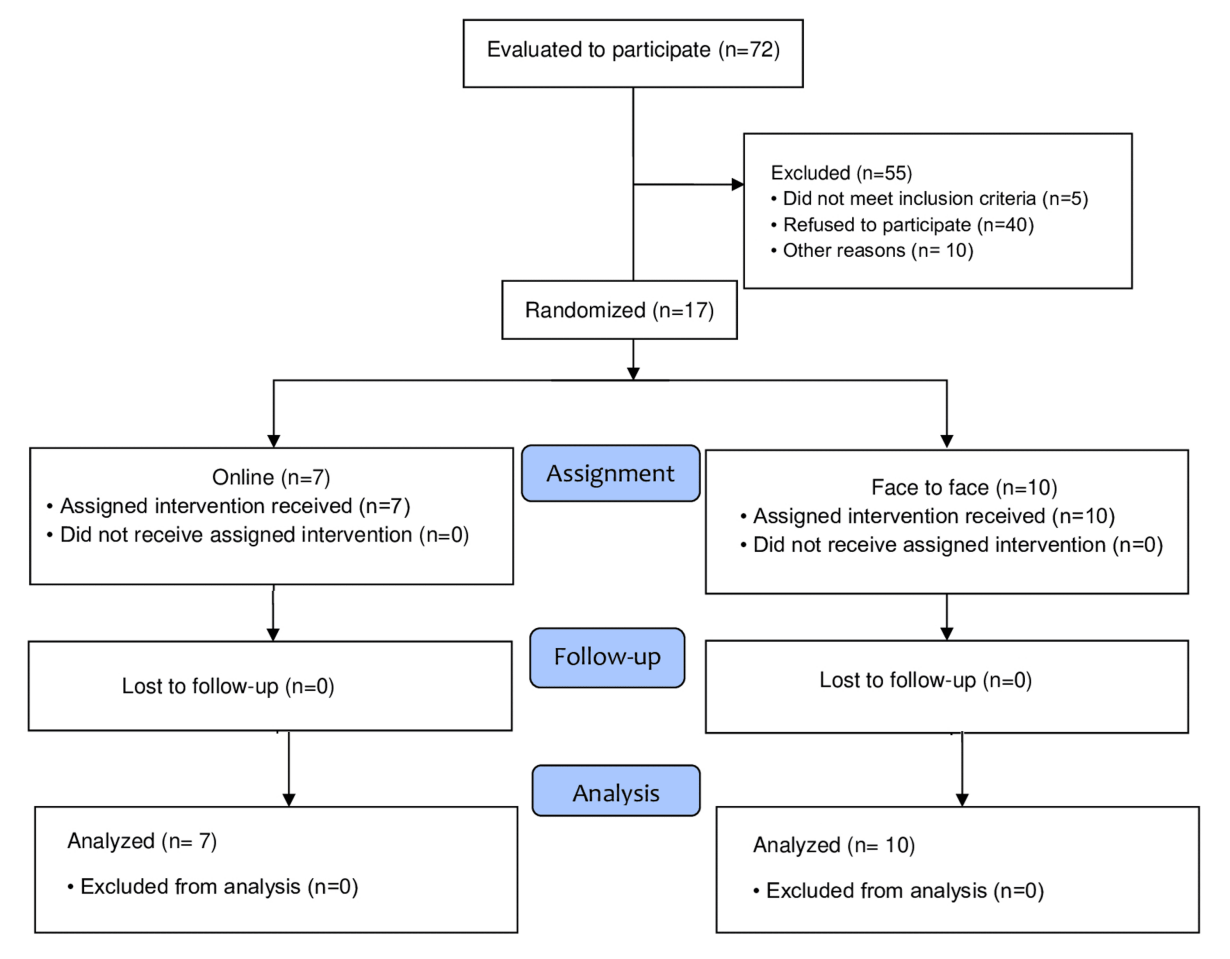
Flowchart of the study process

Patients were randomly assigned to one of the two treatment groups; Figure 1 describes the randomization process and participant follow-up.

The treatment was conducted by two clinical psychologists with a master's degree in cognitive behavioral therapy (CBT) and training in the diagnosis and treatment of insomnia. The techniques implemented in the intervention were Stimulus Control Therapy (SCT), Sleep Restriction Therapy (SRT), Progressive Muscle Relaxation (PMR), and Sleep Hygiene (SH). For a more detailed description, please refer to the Appendices.

Instruments and measurements

Sleep Diary

A sleep diary is the gold standard for recording subjective sleep quality, including sleep duration, naps, number of awakenings per night (Aw), subjective sleep quality (SSQ), and subjective sleep efficiency (SSE) [20].

Pittsburgh Sleep Quality Index (PSQI)

This is a self-administered index with a total score between 0 and 21 points; a score above 5 points is interpreted as poor sleep quality; for Mexican validation, a Cronbach's alpha of 0.78 was obtained [21,22].

Insomnia Severity Index (ISI)

This is a self-administered index with eight items on a Likert scale from 0 (not at all) to 4 (very severe) that assesses nighttime and daytime insomnia symptoms; a reliability coefficient of 0.84 was obtained for Mexican validation [23,24].

Generalized Anxiety Disorder 7 (GAD-7)

This is a self-administered questionnaire that assesses the presence and severity of generalized anxiety symptoms using seven Likert-scale items ranging from 0 (not at all) to 3 (almost every day). The total score ranges from 0 to 21; a total score above 10 is considered generalized anxiety. A version validated for the Mexican population demonstrated high internal consistency, with a Cronbach's alpha of 0.80 [25].

Patient Health Questionnaire 9 (PHQ-9)

This is a self-administered questionnaire that assesses the presence and severity of depressive symptoms using nine Likert-scale items, with a scale from 0 (not at all) to 3 (almost every day). The total score ranges from 0 to 27. These scores are interpreted as mild (total score 0 to 5), moderate (6 to 10), moderately severe (11 to 15), or severe (16 to 27). A Mexican validation obtained a reliability of 0.89 [26].

SF-36 Health Survey

This is a self-administered scale that assesses patients' quality of life. It is divided into eight health-related dimensions, such as physical functioning, role-playing, bodily pain, general health, vitality, social functioning, and emotional and mental health. The first four dimensions are assessed using a Likert scale from 0 to 4; the other four dimensions are assessed using a dichotomous (yes/no) format. A Mexican validation showed high internal consistency (Cronbach's alpha of 0.93) [27].

Procedure

Participants who met the inclusion criteria and agreed to participate provided written informed consent. They then underwent a semi-structured clinical interview conducted by trained research assistants, and the data obtained were added to the database. The randomization sequence was generated using a simple computerized randomization algorithm via the website www.randomizer.org, resulting in a 1:1 allocation ratio between the two treatment groups. The randomization sequence was generated by a statistician (PhD) independent of the study team before participant enrollment. Participant enrollment was performed by the principal investigator, who was unaware of the allocation sequence. Participant allocation to the interventions was performed by a Psychologist (PhD) independent of the research team, who had no involvement in participant enrollment or outcome assessment, thus ensuring allocation blinding. Participants were informed of their group assignment only after completing the initial assessment.

Statistical analysis

Although the Kolmogorov-Smirnov test indicated a deviation from normality, repeated measures ANOVA was retained for the following reasons: first, ANOVA is considered robust to violations of normality when sample sizes are approximately equal across groups and when the number of repeated measurements is limited, as is the case in the present study (three time points: pre-, post-, and follow-up) [28]. Second, the central limit theorem supports the use of parametric tests when deviations from the distribution are moderate rather than severe, particularly in the context of behavioral outcome measures [29]. Third, repeated measures ANOVA allows for the estimation of between-group differences and their 95% confidence intervals (CIs) over time, which is not directly available through fully nonparametric alternatives such as the Friedman test. However, we acknowledge this limitation and complement the parametric approach with Friedman's non-parametric test for within-group comparisons, as well as with effect size estimates (Hedges' G) accompanied by their 95% CIs, thus providing a more complete and interpretable picture of the results.

## Results

A total of 17 participants were included in the study. Sociodemographic variables are presented in Table 1. The age of the BBII-TC group ranged between 20 and 40 years, while the age of the BBII-FF group was 23-40 years (mean = 35.12 (SD = 6.24) vs mean =34.9 (SD = 5.32), p > 0.55). No significant differences were found in most sociodemographic variables except for pharmacological treatment (p < 0.024) (Table 1).

**Table 1:** Sociodemographics, pharmacological treatment, and chronicity of symptoms of the sample (N=17).

Parameters		BBII-TC (n=7), n %	BBII-FF (n=10), n %	Total (N=17), n %	X^2^
Sex	Male	4 (57.1)	6 (60)	10 (58.8)	0.557
	Female	3 (42.8)	4 (40)	7 (41.2)	
Marital Status	Single	4 (57.1)	4 (40)	8 (47.0)	0.464
	Married	2 (28.5)	4 (40)	6 (35.2)	
	Other	1 (14.2)	2 (20)	3 (17.2)	
Educational attainment	High School	0 (0)	7 (70)	7 (41.2)	0.056
	Bachelor’s Degree	7 (100)	3 (30)	10 (58.8)	
Months since the onset of infection	1 - 6	1 (14.2)	1 (10)	2 (11.7)	0.611
	6 - 12	4 (57.1)	2 (20)	6 (35.4)	
	12 - 24	2 (28.5)	7 (70)	9 (52.9)	
Non-psychiatric pharmacological treatment	None	3 (42.8)	7 (70)	10 (58.8)	0.024
	non-psychiatric medication	2 (28.5)	2 (20)	4 (23.6)	
	Other treatment	2 (28.5)	1 (10)	3 (17.6)	
Insomnia duration (months)	1 - 6	4 (57.1)	1 (10)	5 (29.5)	0.263
	6 - 12	3 (42.8)	6 (60)	9 (52.9)	
	12 - 24	0 (0)	3 (30)	3 (17.6)	
Depression duration (months)	1 - 6	6 (85.7)	7 (70)	13 (76.4)	0.24
	6 - 12	1 (14.2)	3 (30)	4 (23.6)	
	12 – 24	0 (0)	0 (0)	0 (0)	
Anxiety duration (months)	1 - 6	5 (71.4)	9 (90)	14 (82.3)	0.92
	6 - 12	2 (28.5)	1 (10)	3 (17.7)	
	12 – 24	0 (0)	0 (0)	0 (0)	

Sleep diary scores and questionnaires

When pooling all subjects, comparing pre, post, and follow-up measurements revealed a significant increase in subjective sleep time (SST), SSQ, and SES. In addition, there was a significant decrease in Aw along with a significant decrease in the ISI, PHQ-9, GAD-7, and PSQI scores (indicating improvement in sleep quality) and mental health, as well as a significant improvement in quality of life (SF-36). All of these changes were maintained to the three-month follow-up (Table 2).

**Table 2:** Comparison, and size of effect in insomnia symptoms, mental health, quality of life and sleep diary between pre-treatment, post-treatment and follow-up. p < 0.05 is considered statistically significant Pre: pre-treatment; Post: post-treatment; FU: follow-up; SST: subjective sleep time; Aw: number of awakenings per night; SSQ: subjective sleep quality; SSE: subjective sleep efficiency; PHQ-9: Patient Health Questionnaire – 9; PSQI: Pittsburgh Sleep Quality Index; ISI: Insomnia Severity Index; GAD-7: Generalized Anxiety Disorder-7; SF-36: Short Form-36; Z: difference between an observed statistic and its hypothetical parameter; b: non-standardized coefficient

Parameters	Pre-Intervention scores	Post-Postintervention scores	3-month follow-up scores	Pre vs Post		Post vs FU		Pre Vs FU	
	Median (rank)	Median (rank)	Median (rank)	Z	p<	Z	p<	Z	p<
SST	4.82 (1.06)	7.58 (2.68)	7.26 (2.26)	-3.644^b^	.000	-3.352^ b^	0.01	-2.441^ b^	(0.00)
SSQ	4.44 (1.00)	8.08 (2.56)	7.82 (2.44)	-3.647^b^	.000	-4.431^ b^	0.01	-3.382^ b^	(0.00)
SSE%	43.35 (1.00)	85.24 (2.74)	79.12 (2.26)	-3.630^b^	.000	-49.658^ b^	0.01	-35.76^b^	(0.00)
Aw	3.76 (2.91)	.06 (1.29)	.53 (1.79)	-3.542^b^	.000	2.542^ b^	0.01	3.235^b^	(0.00)
ISI	14.94 (3.00)	5.94 (1.47)	6.06 (1.53)	-3.626^b^	.000	6.24^ b^	0.01	8.882^b^	(0.00)
PSQI	13.12 (2.94)	4.71 (1.56)	4.59 (1.50)	-3.525^b^	.000	5.719^ b^	0.01	8.529^b^	(0.00)
GAD-7	13.41(2.88)	5.18 (1.56)	5.10 (1.53)	-3.491^b^	.000	5.105^ b^	0.01	8.235^b^	(0.00)
PHQ-9	15.82 (3.00)	4.65 (1.50)	4.65 (1.50)	-3.628^b^	.000	8.511^ b^	0.01	11.176^b^	(0.00)
SF-36	29.65 (1.00)	76.94 (2.47)	77.41(2.53)	-3.627^b^	.000	-55.548^ b^	0.01	-47.76^b^	(0.00)

Subsequently, with an intergroup analysis, we found that in both treatment modalities (BBII-TC vs BBII-FF), there was an increase in the score on the SF-36 and in the SST, SSQ, and SSE; in addition to a significant decrease in the total score of the PHQ-9, PSQI, ISI, GAD-7, and Aw. These changes were also maintained until follow-up, showing that both modalities were effective (Table 3).

**Table 3:** Intergroup mean difference of the intervention modalities. *statistical significance, p < 0.05. SST: subjective sleep time; Aw: number of awakenings per night; SSQ: subjective sleep quality; SSE: subjective sleep efficiency; PHQ-9: Patient Health Questionnaire – 9; PSQI: Pittsburgh Sleep Quality Index; ISI: Insomnia Severity Index; GAD-7: Generalized Anxiety Disorder-7; SF-36: Short Form-36

Parameters	BBII-TC (n = 7)			Differences	BBII-FF (n = 10)			Differences	g Hedges
	Pre Mean (SD)	Post Mean (SD)	Seg. Mean (SD)	p<	Pre Mean (SD)	Post Mean (SD)	Seg. Mean (SD)	p<	
SST	4.85 (1.21)	7.37 (.44)	7.26 (0.66)	-2.384 (.01)	4.80 (1.03)	7.73 (.44)	7.49 (0.45)	-2.820 (.05) *	3.35
SSQ	4.64 (1.43)	7.786 (.80)	7.82 (1.01)	-2.371(.01)	4.30 (.67)	8.30 (.82)	8.10 (0.73)	-2.836 (.05) *	3.91
SSE	50.71(17.89)	87.00 (8.36)	79.12 (7.75)	-2.366(.01)	38.20 (8.03)	84.00 (2.36)	81.00 (5.67)	-2.818 (.05) *	3.90
AW	3.14 (2.54)	0.14 (.37)	0.53 (0.51)	-2.226(.02)	4.20 (.91)	0.00 (.000)	0.60 (0.51)	-2.844 (.05) *	2.91
ISI	14.57 (5.31)	5.57 (1.98)	6.06 (1.92)	-2.37 (.01)	15.20 (3.64)	6.10 (1.88)	6.20(1.98)	-2.812 (.05) *	2.71
PSQI	12.43 (3.55)	4.86 (1.57)	4.59 (1.32)	-2.207 (.02)	13.60 (2.95)	4.60 (1.43)	4.50 (1.23)	-2.807 (.05) *	3.42
GAD-7	13.00 (5.16)	5.29 (3.20)	5.18 (3.08)	-2.388(.01)	13.70 (4.59)	5.10 (3.17)	5.09 (3.05)	-2.603 (.05) *	2.07
PHQ-9	15.86 (3.43)	5.00 (2.38)	4.65 (2.31)	-2.384(0.00)	15.80 (3.45)	4.40 (2.36)	4.41 (2.37)	-2.809 (.05) *	3.88
SF-36	30.00 (2.88)	77.14(9.94)	77.41(11.41)	-2.375 (.01)	29.40 (7.44)	76.80 (12.30)	76.75 (12.30)	-2.810 (.05) *	5.34

Subsequently, a repeated measures ANOVA was carried out to evaluate the intergroup changes. Sleep diary results showed significant within-group improvements from baseline to follow-up in both modalities: SST (F = 97.17; p < 0.01), SSE (F = 175.16; p < 0.01), and SSQ (F = 130.05; p < 0.01) increased significantly, while Aw decreased significantly (F = 55.90; p < 0.01). However, no significant differences were observed between the in-person and online modalities at any follow-up time point. These changes were maintained until follow-up in all five comparisons, and the effect size was medium (Table 4).

**Table 4:** Comparison of sleep diary and sleep scales between the BBII sessions. p < 0.05 is considered statistically significant BBII: Brief Behavioral Intervention for Insomnia; SST: subjective sleep time; Aw: number of awakenings per night; SSQ: subjective sleep quality; SSE: subjective sleep efficiency; PHQ-9: Patient Health Questionnaire-9; PSQI: Pittsburgh Sleep Quality Index; ISI: Insomnia Severity Index; GAD-7: Generalized Anxiety Disorder-7; SF-36: Short Form-36

Parameters	BBII-TC (n = 7)			BBII-FF (n = 10)			p<	Post hoc								
	Pre-treatment score, mean (SD)	Post-treatment, mean (SD)	3-month follow-up, mean (SD)	Pre-treatment, mean (SD)	Post-treatment, mean (SD)	3-month follow-up, mean (SD)										
SST	4.85 (1.21)	7.37 (.44)	7.26 (0.66)	4.80 (1.03)	7.73 (.44)	7.49 (0.45)	0.03	1≤2	2≥3	1=4	1≤5	1≤6	2≤5	2≤6	3≤5	3=6
SSQ	4.64 (1.43)	7.78 (.80)	7.82 (1.01)	4.30 (.67)	8.30 (.82)	8.10 (0.73)	0.98	1≤2	2≤3	1≥4	1≤5	1≤6	2≤5	2≤6	3≤5	3=6
SSE	50.71(17.89)	87.00 (8.36)	79.12 (7.75)	38.20 (8.03)	84.00 (2.36)	81.00 (5.67)	0.00	1≤2	2≥3	1≥4	1≤5	1≤6	2≥5	2≥6	3≤5	3=6
AW	3.14 (2.54)	0.14 (.37)	0.53 (0.51)	4.20 (.91)	.00 (.000)	0.60 (0.51)	0.00	1≥2	2=3	1≤4	1≤5	1≤6	2≥5	2≤6	3≥5	3=6
ISI	14.57 (5.31)	5.57 (1.98)	6.06 (1.92)	15.20 (3.64)	6.10 (1.88)	6.20(1.98)	0.07	1≥2	2≤3	1≤4	1≥5	1≥6	2≤5	2≤6	3≤5	3=6
PSQI	12.43 (3.55)	4.86 (1.57)	4.59 (1.32)	13.60 (2.95)	4.60 (1.43)	4.50 (1.23)	0.07	1≥2	2≤3	1≤4	1≥5	1≥6	2≥5	2≥6	3≤5	3=6
GAD-7	13.00 (5.16)	5.29 (3.20)	5.18 (3.08)	13.70 (4.59)	5.10 (3.17)	5.09 (3.05)	0.05	1≥2	2≥3	1≤4	1≤5	1≤6	2≥5	2≥6	3≥5	3=6
PHQ-9	15.86 (3.43)	5.00 (2.38)	4.65 (2.31)	15.80 (3.45)	4.40 (2.36)	4.41 (2.37)	0.00	1≥2	2≥3	1=4	1≥5	1≥6	2≥5	2≥6	3≥5	3=6
SF-36	30.00 (2.88)	77.14(9.94)	77.41(11.41)	29.40 (7.44)	76.80 (12.30)	76.75 (12.30)	0.00	1≤2	2=3	1≥4	1≤5	1≤6	2≥5	2≥6	3≥5	3=6

Related to sleep disorders and mental health symptoms; significant decreases were found in ISI (F = 77.57, p < 0.01), PSQI (F= 70.96, p < 0.01), GAD-7 (F= 49.44, p < 0.01), and PHQ-9 (F= 125.62, p < 0.01), in addition to a significant increase in SF-36 (F= 200.46, p < 0.01) in both follow-up groups; however there were no significant differences between follow-up in both modalities. The differences were maintained until the follow-up in all five comparisons. In the case of the ISI and the SF-36, the effect size was large. Finally, for the post-hoc results analysis, both groups were divided among the three conditions (pre, post, and follow-up). This resulted in six groups, with groups 1, 2, and 3 being the teleconsultation group and groups 4, 5, and 6 being the face-to-face group (Table 4).

**Figure 2:**
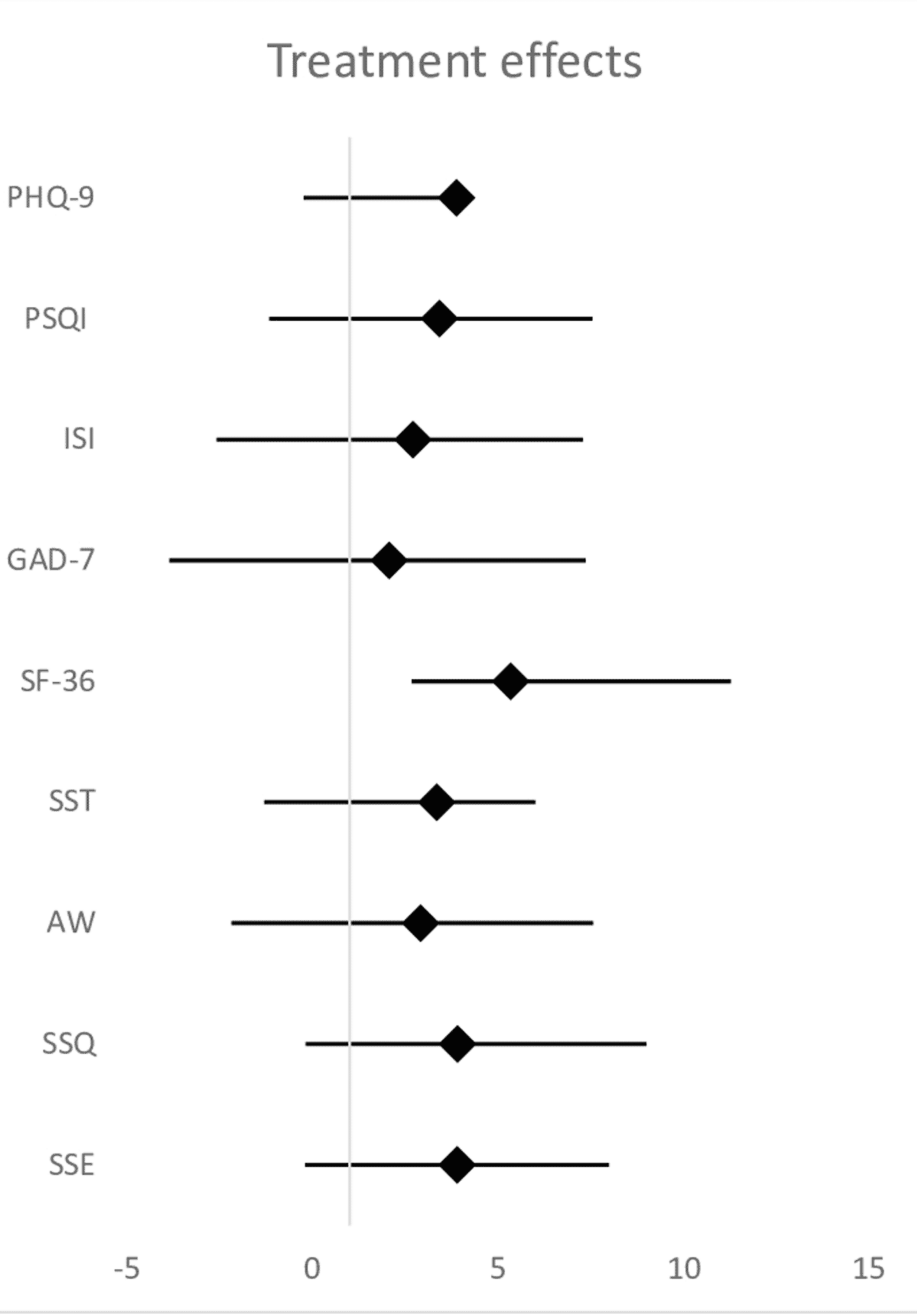
Forest plot BBII: Brief Behavioral Intervention for Insomnia; SST: subjective sleep time; AW: number of awakenings per night; SSQ: subjective sleep quality; SSE%: subjective sleep efficiency; PHQ-9: Patient Health Questionnaire – 9; PSQI: Pittsburgh Sleep Quality Index; ISI: Insomnia Severity Index; GAD-7: Generalized Anxiety Disorder-7; SF-36: Short Form-36

Finally, Figure 2 shows a low effect size for most variables, though changes in quality of life obtained the highest effect size (Hedges g = 5.34).

## Discussion

The objective of this study was to analyze the effectiveness of the BBII in the two administration methods (face-to-face or by telemedicine) on the symptoms of insomnia, anxiety, depression, and quality of life of patients who survived COVID-19 in Mexico City. Considering the advantages of telehealth reported in the literature, it was important to compare telehealth consultation with face-to-face consultation for several reasons. For example, before the COVID-19 pandemic, the prevalence of insomnia in Mexico was estimated at 18.6% [30]; however, there are few sleep disorder clinics in this country, forcing patients to travel to major cities for treatment. In addition, there is no evidence on telehealth for the treatment of insomnia in Mexico. Moreover, telehealth has become an important strategy in the delivery of healthcare, especially since the COVID-19 pandemic [31]. It had become a viable option since Mexican state health systems have the necessary infrastructure to provide adequate care in this area.

Compared to the full CBT-I protocol, BBII was selected because it was less expensive for patients, making it more accessible within a Latin American economic context. Furthermore, European guidelines for the treatment of insomnia state that brief behavioral therapy can be effective on its own [12,32]. Additionally, there are reports demonstrating its effectiveness, and it has been shown to reduce insomnia symptoms such as sleep-onset latency and waking after sleep onset. In some cases, it has resulted in complete remission of insomnia [33]. It has also been reported to improve insomnia severity, sleep latency, sleep efficiency, sleep quality, daytime sleepiness, and the mental components of quality of life, both immediately and after one month [34].

Both treatment modalities (face-to-face and online) resulted in a significant increase in sleep duration, SSQ, and SES to a healthy sleep level, as well as a decrease in insomnia symptoms, mental comorbidity, and an improvement in quality of life (to levels of good mental health and quality of life). In both intervention groups, the ISI and PSQI showed the greatest improvement, with patients who initially presented with moderate clinical insomnia and poor sleep quality achieving insomnia levels below clinical relevance and good sleep quality by the final session [35,36].

Regarding the intervention components, it is important to mention that Sleep Hygiene Education (SHE) is included in this brief behavioral intervention for insomnia. SHE aims to promote better sleep practices by providing information on environmental and lifestyle factors that can modify and reduce the risk of insomnia [37] by controlling factors that perpetuate it. However, SHE has been shown to have limited efficacy as monotherapy, and there is evidence indicating lower therapeutic efficacy compared to CBT-I [38]. The limited efficacy of sleep hygiene as monotherapy may be related to a lack of adaptation to specific ecological and cultural contexts; for example, people living in rural communities are less exposed to artificial nighttime lighting, and in some cases, communities have limited internet access compared to those living in urban areas [39]. In this context, it would be important to compare the efficacy of different sleep hygiene recommendations adapted to particular ecological and cultural environments [40].

When all participants were analyzed together, both treatment modalities were effective at the end of the intervention and during the three-month follow-up, and the effects of the intervention could be expected to last even longer, although further follow-up would be required to confirm the duration of the effects. These findings are consistent with the results of other systematically reviewed clinical trials that have demonstrated the effectiveness of the teleconsultation modality in improving subjective sleep parameters, such as SSQ [41].

Regarding anxiety and depression symptoms, participants' scores on the PHQ-9 and GAD-7 decreased to levels that were not clinically relevant; this is consistent with other studies that have found that anxiety and depression symptoms improve when sleep quality is improved [6]. In this regard, a reciprocal relationship between the HPA axis and sleep has been reported, showing that elevated morning cortisol levels are a significant predictor of subsequent depression [42]; it is possible that the BBII contributed to reducing morning cortisol levels among the patients who participated in this study, thus decreasing anxiety and depression symptoms. This hypothesis warrants further investigation.

It has been previously reported that insomnia treatment is associated with an improvement in quality of life [43]; indeed, in this study, the parameter that showed the most significant improvement (i.e., the largest effect size) was quality of life. This demonstrates the importance of interventions for insomnia. The other variables assessed showed moderate effect sizes. Therefore, our findings are consistent with the literature, indicating that psychological intervention should be the first-line treatment for insomnia, with or without psychiatric comorbidities. Furthermore, it would be important for future research to analyze other variables associated with stress coping, emotional regulation, level of hyperarousal, and neuroticism [44].

In agreement with other studies [45], we observed that face-to-face and online treatment modalities showed no significant differences when compared. However, improvement has been identified as being maintained long-term with the face-to-face modality [46]. Therefore, it would be interesting to determine whether treatment effectiveness differs when comparing the online modality with hybrid modalities (which begin face-to-face and continue online or vice versa) or with mobile or fully digital interventions such as the Sleepio platform [47], the Calm app [48], or the Sleep Ninja smartphone app [49].

Previous studies conducted by other research groups using asynchronous modalities have reported improvements in total sleep time, sleep quality, and sleep efficiency. These results were evaluated both objectively and subjectively, and the findings indicated moderate efficacy at the one-month follow-up [50]. These same results have been observed with teleconsultation modalities [51,52], supporting the initial hypothesis of this study, which states that digital modalities show a similar improvement to face-to-face therapy in insomnia symptoms, as well as in anxiety, depression, and quality of life.

Generalization

Regarding the generalizability of the results, it should be noted that, although the sample consisted of individuals with a medical history of COVID-19 diagnosed by PCR or antigen test, the components of the BBII applied in this study could be applied to patients with insomnia without such a history. It would be important to examine the intervention in larger clinical populations, with a waiting-list control group, and to study more specific variables, such as age, sex, and whether data on neuroticism or hyperarousal exist, to determine the potential generalizability of these findings.

Limitations

Since this was a pilot study, the sample size was small, so future research should increase it to reduce the risk of a Type I error. It will also be important to study the persistence of changes by extending the study duration to include multiple follow-up assessments. Similarly, another important point would be to integrate polysomnographic measures to reduce the subjective aspect of sleep diaries.

Furthermore, it is worth noting that four patients (two from each group) were receiving non-psychiatric medication, which presents a methodological challenge, as some of these drugs are known to influence insomnia symptoms. However, all participants sought medical attention for insomnia; this is important because the intervention is associated with improvements in insomnia and the assessed mental health symptoms, regardless of whether or not they were receiving any type of pharmacological treatment before the intervention.

## Conclusions

Both in-person and virtual modalities of the BBII proved effective in treating insomnia and associated mental health symptoms in patients who survived COVID-19. It is important to note that, despite limitations, encouraging results were found for continuing to adapt treatments for telehealth in developing countries.

## Appendices

Content of the sessions (BBII-TC and BBII-FF)

Session 1. Psychoeducation and Sleep Hygiene

At the beginning of the session, the sleep diary was reviewed, and any difficulties encountered in completing it were discussed. The objectives of the session were then explained. The main topics were Psychoeducation and Sleep Hygiene (causes of insomnia and dysfunctional habits associated with insomnia). Participants were given a sheet with the 10 healthy sleep habits recommended by the World Sleep Society.

1.      Maintain a regular sleep schedule.

2.      Don't take naps longer than 45 minutes.

3.      Don't drink alcohol before bed.

4.      Avoid coffee, tea, or energy drinks.

5.      Avoid heavy meals before bed.

6.      Exercise regularly.

7.      Wear comfortable clothing.

8.      Keep your bedroom at a comfortable temperature and with pleasant lighting.

9.      Don't sleep near noisy sources.

10.  Avoid doing things that keep you awake in bed.

Once the explanation was finished, the sleep diary was analyzed with the participant to implement the sleep restriction technique (to determine the personalized bed schedule). This concluded the first session.

Session 2. Stimulus Control Therapy and Progressive Muscle Relaxation

The session began with the participant's sleep quality progress in the sleep diary, and they were asked to mention the benefits and difficulties they had with the implemented techniques. Later, they were given feedback to overcome the difficulties, and the progress made was reinforced. Then it was explained to them that in this session, a new technique called Stimulus Control Therapy would be implemented, which aimed to modify all the activities that interfered with the onset and maintenance of sleep.

First, it was explained that there were habits that promoted being awake in the bedroom, then dysfunctional behaviors were identified, and finally, their activities in the bedroom were restricted to sleeping or having sex.

Later, it was explained that one of the factors associated with insomnia is stress or anxiety before going to bed, so another technique called Progressive Muscle Relaxation would be taught, the objective of which was to teach the patient to induce sleep. To do this, they were given an information leaflet containing general instructions, and the therapist performed the exercise to give an example of its application. This concluded the second session.

Sessions 3-4. Follow-up and Completion of Treatment

In the last two sessions, the progress obtained in the sleep diary was reviewed, and an evaluation of the objectives set at the beginning of the treatment was made. Finally, the participant were asked to answer the various instruments to obtain data on their progress, and the treatment was concluded.
